# Mechanotransduction *via* a TRPV4-Rac1 signaling axis plays a role in multinucleated giant cell formation

**DOI:** 10.1074/jbc.RA120.014597

**Published:** 2020-12-04

**Authors:** Rakesh K. Arya, Rishov Goswami, Shaik O. Rahaman

**Affiliations:** Department of Nutrition and Food Science, University of Maryland, College Park, Maryland, USA

**Keywords:** TRPV4, macrophages, foreign body response, giant cell, cell fusion, Rac1, AFM, atomic force microscopy, BMDM, bone marrow–derived macrophage, FBGC, foreign body giant cell, FBR, foreign body response, FBS, fetal bovine serum, GFP, green fluorescent protein, GM-CSF, granulocyte macrophage–colony stimulating factor, IL-4, interleukin-4, IP, immunoprecipitation, KO, knockout, PLA, proximity ligation assay, TRPV4, transient receptor potential vanilloid 4

## Abstract

Multinucleated giant cells are formed by the fusion of macrophages and are a characteristic feature in numerous pathophysiological conditions including the foreign body response (FBR). Foreign body giant cells (FBGCs) are inflammatory and destructive multinucleated macrophages and may cause damage and/or rejection of implants. However, while these features of FBGCs are well established, the molecular mechanisms underlying their formation remain elusive. Improved understanding of the molecular mechanisms underlying the formation of FBGCs may permit the development of novel implants that eliminate or reduce the FBR. Our previous study showed that transient receptor potential vanilloid 4 (TRPV4), a mechanosensitive ion channel/receptor, is required for FBGC formation and FBR to biomaterials. Here, we have determined that (a) TRPV4 is directly involved in fusogenic cytokine (interleukin-4 plus granulocyte macrophage–colony stimulating factor)–induced activation of Rac1, in bone marrow–derived macrophages; (b) TRPV4 directly interacts with Rac1, and their interaction is further augmented in the presence of fusogenic cytokines; (c) TRPV4-dependent activation of Rac1 is essential for the augmentation of intracellular stiffness and regulation of cytoskeletal remodeling; and (d) TRPV4-Rac1 signaling axis is critical in fusogenic cytokine–induced FBGC formation. Together, these data suggest a novel mechanism whereby a functional interaction between TRPV4 and Rac1 leads to cytoskeletal remodeling and intracellular stiffness generation to modulate FBGC formation.

Multinucleated giant cells formed by the fusion of macrophages are a hallmark of various chronic inﬂammatory conditions like rheumatoid arthritis, sarcoidosis, and tuberculosis and are associated with the presence of a foreign body or implant in the host (1, 2, 3, 4, 5, 6, 7, 8). Implantation of biomaterials and devices often provoke a foreign body response (FBR), which is an inflammatory reaction in the host tissue that can lead to implant failure, tissue injury, and death of the patient (9, 10, 11, 12, 13). A distinctive feature of the FBR is the accumulation and fusion of macrophages to form destructive and inflammatory multinucleated foreign body giant cells (FBGCs) at the tissue–implant interface (9, 10, 11, 12, 13, 14, 15). Macrophages are recognized as key mediators of the FBR; they respond rapidly to implants, interact with the implant and extracellular matrix, and try to engulf and/or degrade the implant by phagocytosis (16, 17). However, if the implant is too large to engulf, macrophages fuse to form FBGCs and release mediators of degradation such as reactive oxygen species, enzymes, and acid at tissue–implant interfaces that may lead to implant failure (15, 16, 17, 18). Despite the recognized role of macrophages in the FBR, the molecular mechanism underlying FBGC formation remains elusive. Thus, a key objective in the design of future biocompatible implants is to figure out how to regulate macrophage activity without triggering FBR and FBGC generation.

Several cell surface molecules have been linked to FBGC formation. These surface molecules include E-cadherin, mannose receptor, CD44, CD47, CD200, CD36, signal regulatory protein 1-a, IL-4 receptor, and transient receptor potential vanilloid 4 (TRPV4) (9, 10, 11, 12, 19). Additional studies have shown that various cellular proteins or secretory molecules including DAP12, DC-STAMP, Rac1, STAT-6, MMPs, MCP-1, plasma fibronectin, osteopontin, and Prostaglandin E2 and its receptor EP2 modulate macrophage fusion in the formation of FBGCs (19, 20, 21, 22, 23, 24, 25, 26, 27, 28, 29). Besides the role of these molecules required for FBGC formation, emerging studies show that macrophage fusion depends on the chemical composition of the implant, implant stiffness, and extracellular matrix stiffness (30, 31, 32).

TRPV4, a mechanosensitive Ca^2+^-permeable channel, is a member of transient receptor potential superfamily, which is expressed by a broad range of cell types including macrophages (19, 33, 34, 35, 36, 37, 38, 39, 40, 41, 42, 43, 44, 45). Previous studies by our group and others have shown that TRPV4 can be activated by numerous mechanical and biochemical stimuli including shear stress, osmolarity, temperature, and growth factors, as well as by alterations in matrix stiffness *in vitro* and *in vivo* (19, 33, 34, 35, 36, 37, 38, 39, 40, 41, 42, 43, 44, 45). Recently, we reported a novel role of TRPV4 in biomaterial-induced FBRs and FBGC generation (19). The objective of our current study is to determine the mechanism by which TRPV4 modulates FBGC formation.

Cytoskeletal remodeling modulates numerous pathophysiological processes including cell fusion (26, 46, 47). Interestingly, intracellular stiffness (or rigidity) is primarily regulated by cytoskeletal remodeling processes such as F-actin formation (46, 47). It is well recognized that RhoA, Rac1, and Cdc42 small GTPases play important roles in mechanotransduction and in the regulation of many pivotal cellular functions including cellular motility, phagocytosis, cell-to-cell adhesions, and cell-to-extracellular matrix adhesions, the latter being crucial for the formation of multinucleated giant cells (48, 49, 50, 51, 52, 53, 54, 55, 56). Moreover, it has been reported that cytokine-stimulated Rac1 activation is required for lamellipodia formation and subsequent FBGC formation (22). The importance of Rac1 in FBGC formation is further supported by studies reporting that MMP14 forms a complex with CD44, colocalizes with the actin cytoskeleton, and activates Rac1 in the lamellipodia, which is responsible for macrophage migration and fusion (57, 58, 59). Interestingly, in a different cell type, we showed that TRPV4 regulates transforming growth factor–induced F-actin generation as well as activation of RhoA (37). Findings of these studies in concert with our recent findings showing a role of TRPV4 in FBR/FBGC generation (19) suggest the hypothesis that TRPV4 is involved in fusogenic cytokine (interleukin-4 (IL-4) plus granulocyte macrophage–colony stimulating factor (GM-CSF))–induced activation of small Rho GTPase in macrophages and consequent FBGC formation. Here, we report that TRPV4 is indispensable for fusogenic cytokine–induced Rac1 activation but not for RhoA or Cdc42 activation. Intriguingly, we also found that the TRPV4-Rac1 signaling axis is linked to fusogenic cytokine–induced increased macrophage stiffness, lamellipodia/filopodia generation, and FBGC formation.

## Results

### Fusogenic cytokine–induced Rac1 activation is reliant on TRPV4

**Figure 1:**
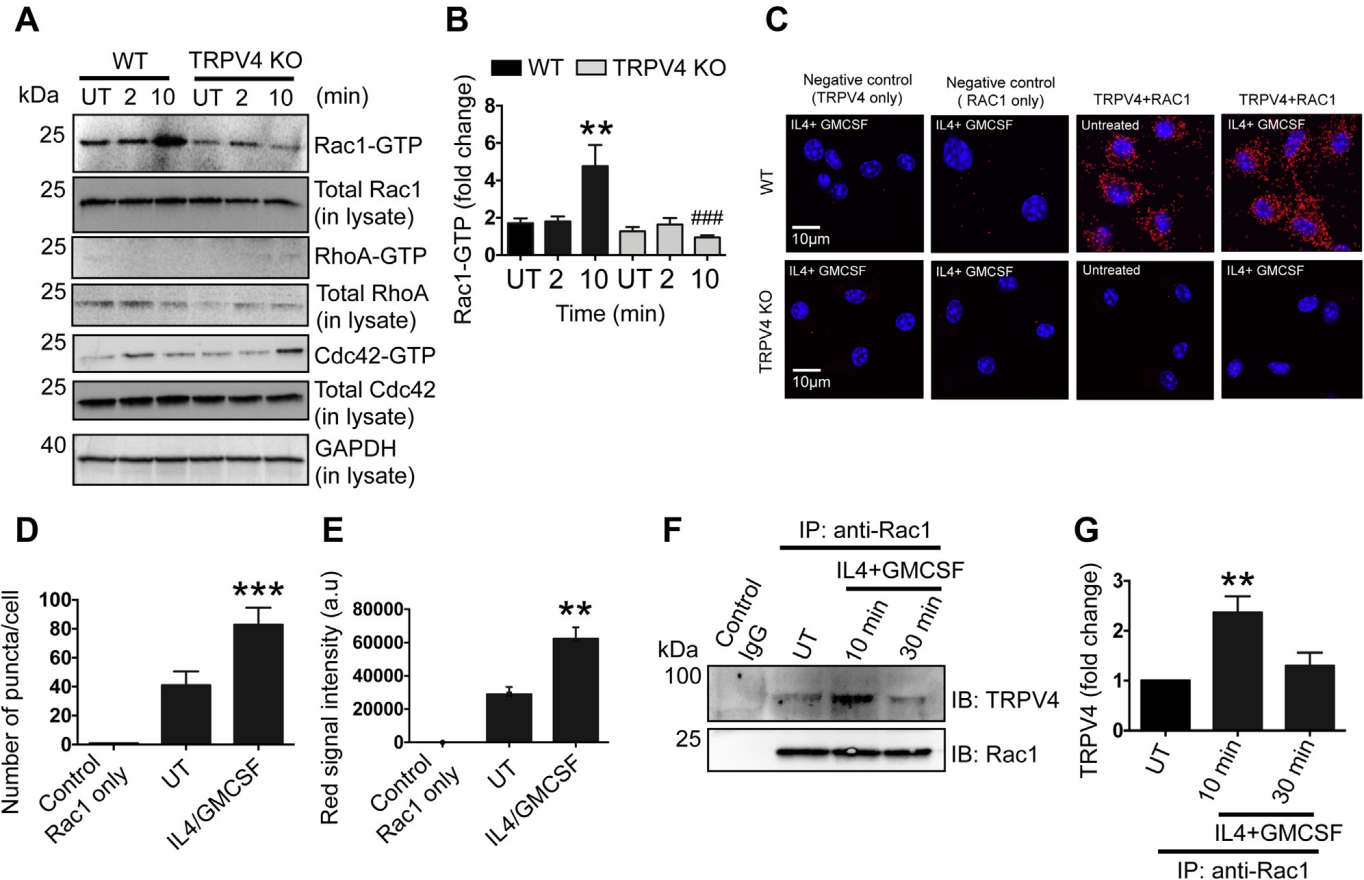
**TRPV4 selectively activates Rac1 in BMDMs stimulated by IL-4 plus GM-CSF and interacts directly with Rac1.***A*–*B*, BMDMs from WT or TRPV4 mice were stimulated with IL-4 plus GM-CSF (25 ng/ml) for 0, 2, and 10 min, and whole-cell lysates were prepared for the analysis of total and activated Rac1, RhoA, and Cdc42. *A*, immunoblots showing expression levels of activated Rac1, RhoA, and Cdc42 in whole-cell lysates after antibody-mediated pull down. Total RhoA, Rac1, and Cdc42, as well as GAPDH, were analyzed in whole-cell lysates. Results are representative of three independent experiments. *B*, quantification of results from experiment shown in A. Student’s *t* test; ∗∗*p* < 0.01 (0 min *versus* 10 in WT), ###*p* < 0.001 (10 min in WT *versus* 10 min in TRPV4 KO). *C*, interaction between TRPV4 and Rac1 proteins was determined using proximity ligation assay in unstimulated or IL-4 plus GM-CSF–stimulated (10 min) WT and TRPV4 KO BMDMs. WT and TRPV4 KO BMDMs with no primary antibodies were used as a negative control for the assay. Images (original magnification: 63× oil) are representative of five different fields per condition. *D*–*E*, quantification of data from the experiment shown in C. *D*, bar graph shows the TRPV4–Rac1 interaction efficiency as the number of red puncta per cell in different conditions. *E*, histograms show quantification of red signal intensity per field under different conditions. Student’s *t* test, n ≥ 3 independent experiments, ∗∗*p* ≤ 0.01, ∗∗∗*p* ≤ 0.001. *F*, co-immunoprecipitation (IP) followed by immunoblot (IB) analysis shows interaction of TRPV4 with Rac1 in IL-4 plus GM-CSF–stimulated macrophages at 10 and 30 min. Isotype control IgG was used as a control. *G*, quantification of results from experiment shown in *F*. Student’s *t* test; n = 3 independent experiments, ∗∗*p* < 0.01 (UT *versus* 10 min in WT macrophages). BMDM, bone marrow–derived macrophage; GM-CSF, granulocyte macrophage–colony stimulating factor; IL-4, interleukin-4; TRPV4, transient receptor potential vanilloid 4.

We recently reported that TRPV4 is required for FBR and multinucleated FBGC formation (19). Small Rho family GTPases have a well-recognized role in cytoskeletal remodeling and in actin filament dynamics, which are reported to play a role in multinucleated giant cell formation (53, 54, 55, 56). We asked whether TRPV4 modulated FBGC formation *via* activation of Rho GTPases by determining the activity of the three well-recognized small GTPases (RhoA, Rac1, and Cdc42) in whole-cell lysates of fusogenic cytokine (IL-4 plus GM-CSF)–induced bone marrow–derived macrophages (BMDMs) from WT and TRPV4 knockout (KO) mice at two different time points. Using a glutathione s-transferase bead–based pull-down assay, we found a specific and significant upregulation of activated Rac1 (Rac1-GTP) after 10 min of stimulation with IL-4 plus GM-CSF in WT cells but not in TRPV4 KO cells (Fig. 1, *A*–*B*). However, the levels of active RhoA (RhoA-GTP) and Cdc42 (Cdc42-GTP) in pull-down lysates and of all three total GTPases in whole-cell lysates did not change significantly or remained unchanged after IL-4 plus GM-CSF stimulation in both WT and TRPV4 KO cells (Fig. 1*A*). These results suggest that IL-4 plus GM-CSF–induced specific activation of Rac1 was dependent on TRPV4. Next, we performed proximity ligation assays (PLAs) to directly test whether TRPV4 and Rac1 physically interact with each other in BMDMs under IL-4 plus GM-CSF stimulation. PLA, an antibody-based immunohistochemical method, allows detection of protein–protein interactions by *in situ* detection of proteins in close proximity (30–40 nm apart) with high specificity and sensitivity (60). Using PLA, we found distinct fluorescent red puncta in unstimulated WT cells, suggesting an interaction of TRPV4 with Rac1 under basal conditions (Fig. 1, *C*–*E*). After stimulation with IL-4 plus GM-CSF, interaction of TRPV4 with Rac1 in BMDMs increased by 2-fold as indicated by the increased total number of puncta/cell and fluorescence intensity; specificity of the antibody was shown by the lack of puncta in the no-primary-antibody control in WT and TRPV4 KO cells (Fig. 1, *C*–*E*). In addition, we performed co-immunoprecipitation (IP) assay to directly test whether TRPV4 and Rac1 interact with each other in BMDMs in response to IL-4 plus GM-CSF stimulation. Using IP, we found that after stimulation with IL-4 plus GM-CSF, interaction of TRPV4 with Rac1 in BMDMs increased (Fig. 1, *F*–*G*). All together, these results suggest a direct interaction of TRPV4 with Rac1 in BMDMs.

### TRPV4 is directly involved in fusogenic cytokine–induced activation of Rac1 in macrophages

**Figure 2:**
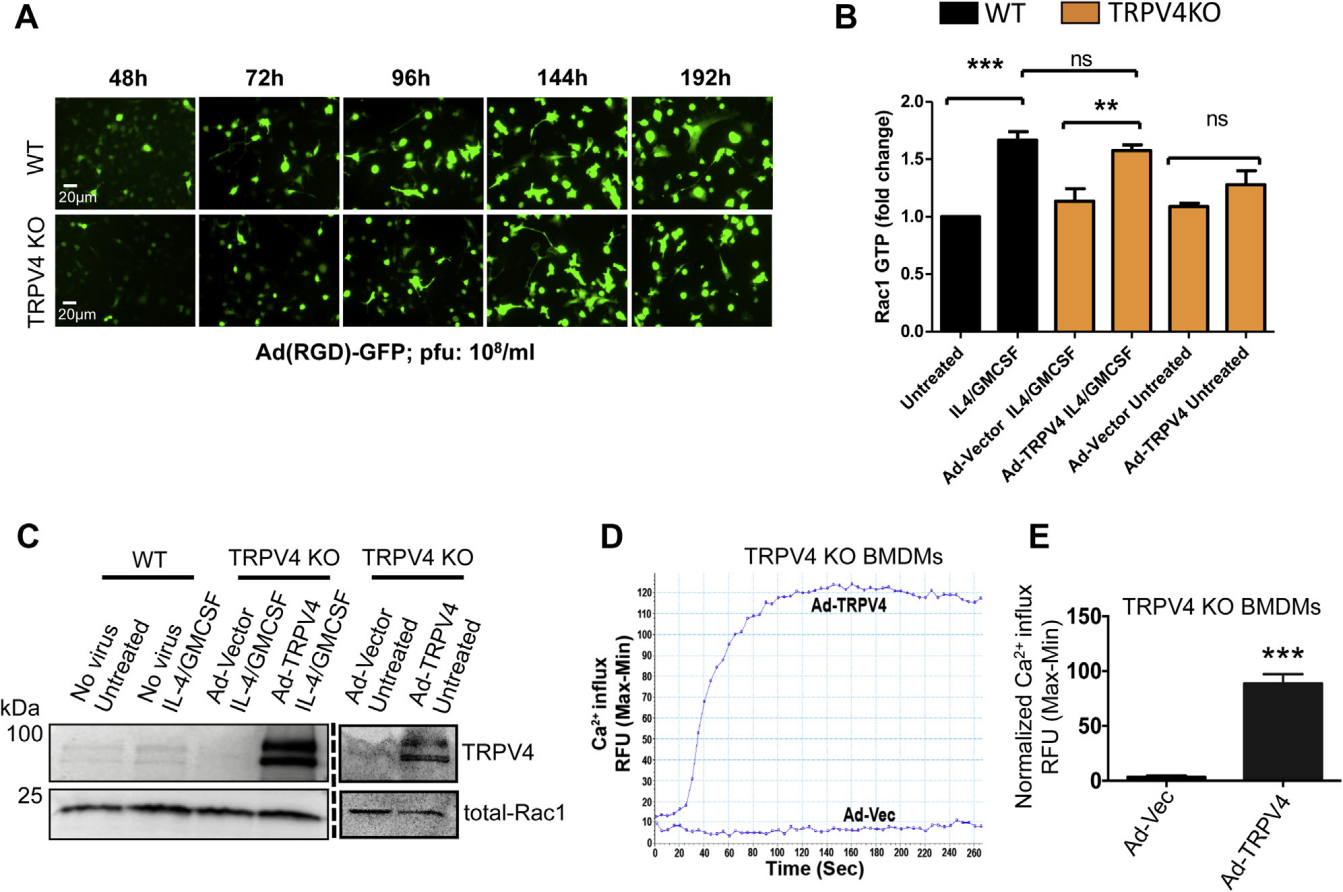
**TRPV4 directly regulates IL-4 plus GM-CSF–induced activation of Rac1 in macrophages**. *A*, BMDMs from WT and TRPV4 KO mice were transduced with Ad(RGD)-GFP (1 × 10^8^ pfu/ml). The expression and retention time of Ad(RGD)-GFP vector was confirmed in BMDMs by fluorescence microscopy at different time points. Representative images are shown; 20× magnification. *B*–*C*, WT BMDMs were treated with/without IL-4 plus GM-CSF, and TRPV4 KO BMDMs were transduced with either Ad-Vec or Ad-TRPV4 and were untreated or treated with IL-4 plus GM-CSF for 10 min on day 5 of transduction. *B*, Rac1-GTP levels were determined by G-LISA assay. The expression level was normalized to the total Rac1 level in each sample. Data are expressed as mean ± SEM, n = 3 independent experiments, Student’s *t* test, ∗∗*p* < 0.01, ∗∗∗*p* < 0.001, *ns*, not significant. *C*, TRPV4 and total Rac1 expression levels in both transduced and untransduced cells were analyzed by Western blotting. *D*, FlexStation 3 recording of GSK1016790A-induced Ca^2+^ influx in TRPV4 KO BMDMs transfected with Ad-TRPV4 or Ad-Vec constructs. *E*, bar graph shows quantification of Ca^2+^ influx from the experiment. The experiment was repeated three times in quadruplicate. Student’s *t* test, ∗∗∗*p* < 0.001. BMDM, bone marrow–derived macrophage; GM-CSF, granulocyte macrophage–colony stimulating factor; IL-4, interleukin-4; RFU, relative fluorescence unit; TRPV4, transient receptor potential vanilloid 4.

To assess whether TRPV4 is directly involved in Rac1 activation, we used a gain-of-function approach by overexpressing Ad-TRPV4 in TRPV4 KO macrophages. We first determined the transduction efficiency and sustainability of adenovirus constructs in BMDMs by examining Ad-(RGD)-green fluorescent protein (GFP) expression in WT and TRPV4 KO BMDMs at different time points. We found that GFP expression was maximal after 4 days and was sustained up to 8 days after transduction (Fig. 2*A*). To assess the direct involvement of TRPV4 in activation of Rac1, we transduced TRPV4 KO BMDMs with either Ad-TRPV4 or Ad-Vec. After 4 days of transduction, cells were treated with IL-4 plus GM-CSF for 10 min, and intracellular Rac1-GTP was measured by G-LISA following a standard protocol. For controls, we determined intracellular Rac1-GTP from WT BMDMs with or without IL-4 plus GM-CSF treatment. Results showed significant activation of Rac1 after IL-4 plus GM-CSF treatment in TRPV4 KO BMDMs with TRPV4 overexpression that was comparable with the Rac1 activation in IL-4 plus GM-CSF–induced WT BMDMs (Fig. 2*B*). However, the activation of Rac1 after IL-4 plus GM-CSF treatment in Ad-Vec–transduced TRPV4 KO BMDMs was similar to that in the untreated WT BMDMs (Fig. 2*B*). Together, these results showed an absolute requirement for TRPV4 in fusogenic cytokine–induced activation of Rac1 in macrophages. We confirmed the functional expression of TRPV4 by adenovirus constructs in TRPV4 KO BMDMs by immunoblot analysis (Fig. 2*C*) and by induction of Ca^2+^ influx by a TRPV4-specific agonist (Fig. 2, *D*–*E*).

### TRPV4-Rac1 signaling axis plays a critical role in the augmentation of intracellular stiffness and cytoskeletal remodeling under fusogenic conditions

**Figure 3:**
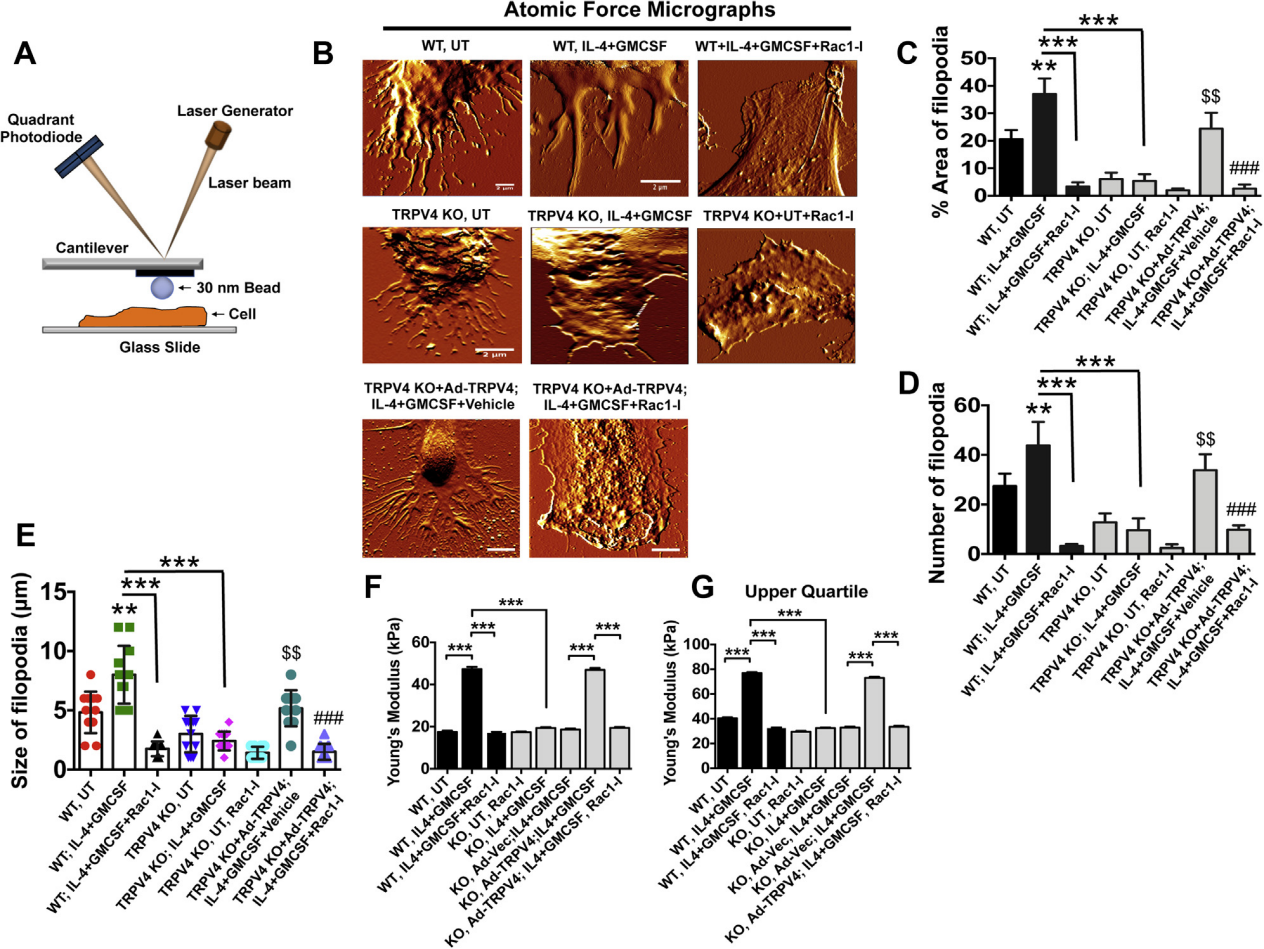
**TRPV4-Rac1 signaling axis plays a crucial role in the augmentation of intracellular stiffness and regulation of cytoskeletal remodeling in BMDMs**. *A*, schematic diagram of atomic force microscopy (AFM) setup to determine the stiffness (Young’s modulus) of BMDMs. A detector records deflection of a laser beam by deformation of the cantilever attached to a circular symmetric quartz probe with a radius of 30 nm. Force curves generated by this process are fitted to the Hertz model to achieve Young’s modulus value (kPa). *B*, representative high-resolution AFM micrographs show distribution of lamellipodia/filopodial areas of indicated cell groups. Scale bars: 2 μm; n = 10 cells/group; 2 scanned areas/cell. Quantification of data from experiment shown in B: Histograms show percent area of filopodia (*C*), the number of filopodia (*D*), and size of filopodia (*E*). Student’s *t* test; $$*p* < 0.01 (KO, UT *versus* KO+Ad-TRPV4), ∗∗*p* < 0.01 (WT, UT *versus* WT+IL-4+GM-CSF), ∗∗∗*p* < 0.001, and ###*p* < 0.001 (KO+Ad-TRPV4 *versus* KO+Ad-TRPV4+Rac1-I). *F*, quantification of Young’s modulus (kPa) of the dataset, and *G*, upper quartile data points acquired from the experiment shown in B. n = 70 data points/group; One-way ANOVA followed by Bonferroni test; ∗∗∗*p* < 0.001. BMDM, bone marrow–derived macrophage; GM-CSF, granulocyte macrophage–colony stimulating factor; IL-4, interleukin-4; TRPV4, transient receptor potential vanilloid 4.

Cytoskeletal remodeling regulates numerous physiological processes including cell fusion (26, 46). Previous reports showed that Rac1 plays a crucial role in lamellipodia and filopodia formation and in macrophage fusion (22). Interestingly, intracellular stiffness is predominantly regulated by F-actin generation, a cytoskeletal remodeling process (47, 48, 49). In view of our observation that TRPV4 was required for fusogenic cytokine–induced activation of Rac1, we sought to determine if the TRPV4-Rac1 signaling axis was directly involved in intracellular stiffness induction and cytoskeletal remodeling in macrophages under fusogenic conditions. Using high-resolution atomic force microscopy (AFM) imaging, we found that TRPV4 deletion impaired lamellipodia/filopodia formation in TRPV4 KO BMDMs compared with WT as evidenced by the decreased number, size, and area of filopodia (Fig. 3, *A*–*E*). Reconstitution of TRPV4 KO BMDMs with Ad-TRPV4 followed by fusogenic cytokine treatment reversed impaired lamellipodia/filopodia formation compared with untransduced TRPV4 KO BMDMs, suggesting a direct role of TRPV4 in this process (Fig. 3, *B*–*E*). Additional results showed that Ad-TRPV4–dependent reversion of lamellipodia/filopodia formation in TRPV4 KO cells was prevented by treatment with a small-molecule inhibitor of Rac1 (Rac1-I), suggesting that Rac1-supported lamellipodia/filopodia formation was mediated by TRPV4 (Fig. 3, *B*–*E*). We also determined the generation of intracellular stiffness (Young’s module) in BMDMs using AFM under similar conditions as above and found that the stiffness level decreased by 2-fold in the presence of the Rac1 inhibitor in Ad-TRPV4–overexpressing TRPV4 KO BMDMs compared with vehicle-treated Ad-TRPV4–overexpressing TRPV4 KO or WT BMDMs under fusogenic conditions (Fig. 3, *F*–*G*). Upper quartile values followed the same trend, suggesting that differences in stiffness remained the same at the stiffer region under all five experimental conditions (Fig. 3*G*). Taken together, these results suggest that the TRPV4-Rac1 signaling axis is directly involved in intracellular stiffness induction and cytoskeletal remodeling in BMDMs under fusogenic conditions.

### TRPV4-dependent Rac1 activation is essential for FBGC formation

**Figure 4:**
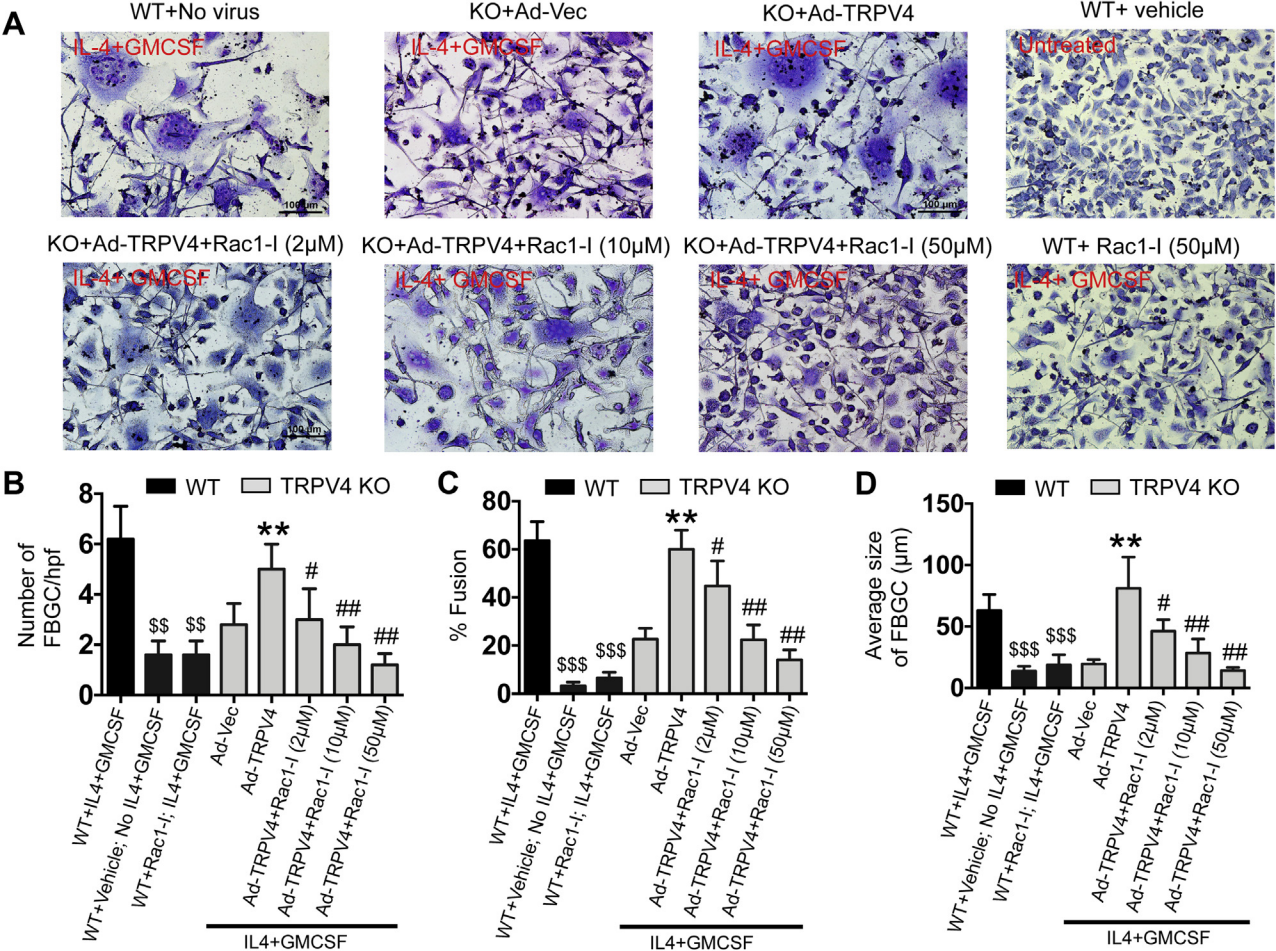
**TRPV4-dependent Rac1 activation regulates fusogenic cytokine–induced FBGC formation.***A*, Giemsa-stained images showing FBGC formation by WT or TRPV4 KO BMDMs transduced with Ad-Vec or Ad-TRPV4 construct with or without Rac1-I (2, 10, and 50 μM) treatment after 8 days of fusogenic cytokine stimulation. Quantification of the number of FBGC/high power field (*B*), percent fusion (*C*), and average size of FBGCs (*D*) from experiment shown in (*A*). Scale bars: 50 μm; Student’s *t* test for *B*–*D*; ∗∗*p* < 0.01 (Ad-Vec *versus* Ad-TRPV4), #*p* < 0.05 (Ad-TRPV4 *versus* Ad-TRPV4+Rac1-I), ##*p* < 0.01, ###*p* < 0.001, $$*p* < 0.01, and $$$*p* < 0.001 (WT, IL-4+GM-CSF *versus* WT+ No IL-4+GM-CSF or WT+Rac1-I+IL-4+GM-CSF). BMDM, bone marrow–derived macrophage; FBGC, Foreign body giant cell; GM-CSF, granulocyte macrophage–colony stimulating factor; IL-4, interleukin-4; TRPV4, transient receptor potential vanilloid 4.

Since generation of both cytoskeletal remodeling and matrix stiffness was found to be associated with cell fusion, and both processes were found to be dependent on the TRPV4-Rac1 axis, we assessed the dependence of TRPV4-induced FBGC formation on activation of Rac1. TRPV4 KO BMDMs overexpressing Ad-TRPV4 showed an increase in the number of FBGC, as well as an increase in the percentage of fusion and in the average size of FBGCs compared with the Ad-Vec–transduced counterparts; these factors were all significantly diminished in a dose-dependent manner by the presence of the Rac1 inhibitor (Fig. 4, *A*–*D*). Additional results showed that IL-4 plus GM-CSF–induced FBGC formation in WT BMDMs was inhibited by treatment with the Rac1 inhibitor, suggesting that endogenous TRPV4 regulates FBGC formation *via* Rac1 (Fig. 4*A*). Altogether, these results suggest that Rac1-dependent remodeling of cytoskeletal structure and consequent induction of intracellular stiffness plays an essential role in TRPV4-dependent FBGC formation.

## Discussion

Cell fusion is an essential process in several pathophysiological conditions including fertilization, development, bone formation and the response to implants (4, 5, 61). We are interested in the molecular mechanisms by which mechanical and soluble signals are integrated to support the development of a biological response. Our recently published data show that TRPV4, a mechanosensitive ion channel/receptor, plays a key role in biomaterial-induced development of FBR and multinucleated FBGC formation (19). We reported that macrophage fusion is dependent specifically on TRPV4-elicited Ca^2+^ influx (19). We used selective small-molecule chemical agonist (GSK1016790A) and antagonist (GSK2193874) that specifically elicits or blocks TRPV4-dependent Ca^2+^ influx, respectively. We found that macrophage fusion in antagonist-treated BMDMs was significantly impaired compared with vehicle-treated control cells. Treatment of BMDMs with the agonist increased the amount of cell fusion compared with vehicle-treated control cells. Our current study sheds light on the molecular mechanism of these effects through several major findings: (a) TRPV4 is directly involved in fusogenic cytokine (IL-4 plus GM-CSF)–induced activation of Rac1, but not RhoA or Cdc42 in BMDMs; (b) TRPV4 physically interacts with Rac1, and their interaction is augmented in response to stimulation with fusogenic cytokines; (c) TRPV4-dependent activation of Rac1 plays a critical role in the augmentation of intracellular stiffness and regulation of cytoskeletal remodeling; and (d) TRPV4-Rac1 signaling is required in fusogenic cytokine–induced FBGC formation.

Our previously published data show a link between activation of Rho GTPases and TRPV4 in lung fibroblasts where TRPV4 upregulation was shown to promote actin polymerization and stress fiber formation through RhoA activation (37). It was also reported that TRPV4 regulates migration and invasion of glioma cells *via* Rac1 signaling (62). Herein, we showed that TRPV4 deficiency specifically abrogated IL-4 plus GM-CSF–induced activation of Rac1 in macrophages, which was restored by TRPV4 reintroduction. Using a proximity ligation assay, we showed interaction of TRPV4 with Rac1 under basal unstimulated conditions, which increased by 2-fold after stimulation with fusogenic cytokines as indicated by an increase in the total number of puncta/cell and in fluorescence intensity. IP assay also showed direct interaction of TRPV4 with Rac1 in BMDMs stimulated by fusogenic cytokines. These results suggest a direct and constitutive interaction of TRPV4 with Rac1 in macrophages.

TRPV4 receptors/channels can sense diverse biomechanical and biochemical stimuli including matrix stiffness and soluble factors and converts/integrates them to a Ca^2+^-dependent signal to induce a cellular response, in part, through modulating the cytoskeletal structure (33, 37, 39, 40, 41, 47, 48, 49, 50, 51, 52). The response to biomechanical or physical stimuli involves force generation that produces cellular protrusions such as lamellipodia, filopodia, and podosomes *via* remodeling the actin cytoskeleton, which consists of monomeric (globular; G-actin) and polymeric (filamentous; F-actin) actin and actin-binding proteins (47, 48, 49, 50, 51). Filopodia are cell extensions made of short bundles of F-actin, which are implicated in making first contacts with opposing cell fronts, where they serve as precursors for the formation of mature cell–cell junctions for cell fusion (26, 46, 50, 51, 52). Since Rac1 plays an important role in mechanotransduction and regulation of many pivotal cellular responses including cytoskeletal remodeling (48, 49, 50, 51, 52, 53, 54, 55, 56), our current finding that fusogenic cytokines induce activation of Rac1 in a TRPV4-dependent manner further suggests a mechanism in which TRPV4-dependent Rac1 activation is associated with fusogenic cytokine–induced cytoskeletal remodeling. Using AFM analysis, we showed that lack of TRPV4 function abrogated fusogenic cytokine–induced cytoskeletal remodeling in BMDMs, and this remodeling was re-established by TRPV4 reintroduction. These results suggest that TRPV4 is absolutely required for cytoskeletal remodeling in BMDMs under fusogenic conditions. Additional results showed that TRPV4 reintroduction–dependent reversion of lamellipodia/filopodia formation in TRPV4 KO cells was blocked by treatment with an Rac1 inhibitor, suggesting that Rac1-supported lamellipodia/filopodia formation was mediated by TRPV4. These results are consistent with a report showing that a direct molecular association of cytoskeletal elements, including actin filaments and microtubules, with TRPV4 and consequent cell morphological changes affecting lamellipodial and filopodial structure (63). Interestingly, intracellular stiffness is predominantly dependent on cytoskeletal remodeling processes such as F-actin generation (47, 48, 49). Using AFM analysis, herein, we showed that TRPV4 deficiency abrogated fusogenic cytokine–induced intracellular stiffness induction in BMDMs, which, again, was re-established by TRPV4 reintroduction. Additional results showed that TRPV4 reintroduction–dependent reversion of intracellular stiffness generation in TRPV4 KO cells was blocked by treatment with an Rac1 inhibitor. Altogether, these results suggest that the TRPV4-Rac1 signaling axis is absolutely required for intracellular stiffness induction in BMDMs under fusogenic conditions possibly *via* cytoskeletal remodeling.

Cell–cell fusion is a vital process in macrophage FBGC formation, an inflammatory and destructive multinucleated cell type associated with implant-induced FBR (1, 2, 3, 4, 5, 6). Cell fusion involves cytoskeletal rearrangement followed by the generation of pseudopodia/filopodia, which, at the forefront of fusion events, contribute to the sharing of cytoplasm between fusing partners (1, 2, 3, 4, 64). Since lamellipodia formation *via* the Rac1 pathway has been previously implicated in macrophage fusion and FBGC formation (22, 65), we assessed the possibility that the mechanism by which TRPV4 mechanosensing modulates FBGC formation is dependent on Rac1. We found that TRPV4 overexpression–mediated reversion of FBGC formation, the percentage of cells undergoing fusion, and the size of FBGCs in TRPV4 KO BMDMs were suppressed by Rac1 inhibitor in a dose-dependent manner, suggesting that Rac1 activation triggers a signal that acts downstream of TRPV4 during macrophage fusion. It has been reported that Rac1 activity and function are regulated by its subcellular distribution and translocation in many cell types (66, 67). Whether similar Rac1 regulation is also required for TRPV4-dependent FBGC formation remains to be determined.

In summary, the results of our study support the notion that a functional interaction between TRPV4 and Rac1 leads to cytoskeletal remodeling and cellular force generation to modulate FBGC formation. As such, our study provides insight into the mechanism of FBGC formation, which might be exploited in the development of immune-competent implants and therapeutics.

## Experimental procedures

### Cytokines and reagents

Rac1, Cdc42, and RhoA Pull-Down Activation Assay Biochem Kits (Bead Pull-Down Format) (which included antibodies against Rac1, Cdc42, and RhoA) and Rac1 G-LISA Activation Assay Kit (Colorimetric Based) were procured from Cytoskeleton (Denver, CO, USA). Antibodies against TRPV4 (Cat# ACC-034, Alomone Labs, Jerusalem, Israel) and control mouse IgG and GAPDH (Santa Cruz Biotechnology, Dallas, TX, USA) were purchased. Giemsa solution, GSK1016970A, and bovine serum albumin (BSA) were purchased from Sigma-Aldrich (St Louis, MO). Goat anti-mouse and goat anti-rabbit secondary IgGs were purchased from Jackson ImmunoResearch (West Grove, PA). Mouse anti-rabbit IgG conformation–specific secondary antibody (L27A9; HRP Conjugate mAb; cat #5127) and light chain–specific rabbit anti-mouse IgG (D3V2A; mAb;HRP Conjugate; cat #58802) were procured from Cell Signaling Technology (MA, USA). Duolink *In Situ* Red Starter PLA Kit Mouse/Rabbit, Rac1 antibody (Cat# 05-389, clone 23A8), protein G-Sepharose beads, and Rac1 inhibitor (Rac1-I) were obtained from Millipore-Sigma (Massachusetts, USA). Macrophage colony–stimulating factor, mouse IL-4, and GM-CSF were obtained from R&D Systems (Minneapolis, MN). FLIPR calcium 6 assay kit for Ca^2+^ influx recording was purchased from Molecular Devices (Sunnyvale, CA, USA). DMEM media, fetal bovine serum (FBS), and cell culture–related reagents were obtained from Gibco. Adenovirus vector expressing mouse-TRPV4, Ad(RGD)-TRPV4-HIS, Ad(RGD)-GFP, and control vector construct Ad(RGD)-CMV-null were obtained from Vector Biolabs (Malvern, PA, USA). All other reagents used were purchased from Sigma-Aldrich or Thermo Fisher Scientific.

### Animal and cell culture

WT C57BL/6 mice were purchased from Charles River Laboratories (Wilmington, Massachusetts, USA). TRPV4 KO (TRPV4 KO or TRPV4^−/−^) mice were originally created on a C57BL/6 background by Dr M Suzuki (Jichi Medical University, Tochigi, Japan); we acquired TRPV4 KO mice from Dr David X. Zhang (Medical College of Wisconsin, Milwaukee, WI, USA) (68, 69). All experiments on mice were conducted in accordance with the Institutional Animal Care and Use Committee guidelines and were approved by the University of Maryland-College Park Review Committee. Murine BMDMs were harvested from 6- to 7-week old mice, as previously described (19, 70, 71, 72). Briefly, femurs from WT and TRPV4 KO mice were collected, and bone marrow of femurs was ﬂushed out with complete DMEM media with 10% FBS. The suspended bone marrow cells were ﬁltered through a 70-μm strainer (BD bioscience), centrifuged, and maintained in DMEM medium supplemented with macrophage colony–stimulating factor (25 ng/ml), for 7 to 8 days to differentiate into macrophages at 37 °C.

### Rho GTPase activity assays, Western blotting, and IP

Rac1, RhoA, and Cdc42 activities were determined using the Pull-Down Activation Assay Biochem Kit following the manufacturer’s instructions. Briefly, BMDMs from both WT and TRPV4 KO mice were maintained in DMEM with 2.5% serum for 72 h and then in serum-free media for additional 24 h, followed by treatment with or without IL-4 plus GM-CSF (25 ng/ml) for 2 min or 10 min. After treatment, cells were lysed using a cell scraper on ice in chilled lysis buffer supplemented with protease inhibitor cocktail provided with the kit. Cell lysates were centrifuged (10,000*g*, 4 °C for 1 min) to remove cell debris, and then lysate (500 μg) was incubated for 60 min with p21-activated kinase-GST beads (15 μg) for Rac1/Cdc42 or Rhotekin-GST beads (50 μg) for RhoA, respectively, at 4 °C to pull down GTP-bound Rac1, Cdc42, or RhoA. Beads were then washed in wash buffer B. The pull-down fractions were run in parallel with total cell lysates in 12% SDS-PAGE under reducing conditions. Proteins were transferred to PVDF membranes and immunoblotted with mouse monoclonal primary antibodies against RhoA, Rac1, or Cdc42 provided with the kit or GAPDH (loading control for whole-cell lysates) followed by secondary horseradish peroxidase–conjugated antibodies. The signals were visualized using an enhanced chemiluminescence system (UVP Biospectrum, Upland, CA, USA). Band intensities were quantified with NIH Image J software, and the relative amount of active, GTP-bound GTPase was normalized to the total content of GTPase in the lysate. To detect TRPV4 in Western blots, cells were lysed in RIPA buffer containing both phosphatase and protease inhibitors. Equal amounts of total protein from each sample were resolved by SDS-PAGE, transferred to a PVDF membrane, and probed with anti-TRPV4. For IP studies, BMDMs were lysed in RIPA buffer. The cleared supernatant containing 500 μg of proteins was incubated with 4 μg of Rac1 or isotype control antibodies immobilized on G-sepharose beads for 2 h at 4 °C. Beads were washed extensively in the RIPA buffer and then boiled in SDS-PAGE loading buffer for subsequent immunoblotting with antibodies for Rac1 and TRPV4. Immunoblots were analyzed with a chemiluminescence detection system.

### *In situ* PLA

BMDMs from WT mice were seeded on Permanox plastic slides (Lab-Tek chamber slides; Nunc, Grand Island, NY, USA) at a density of 1 × 10^5^ cells per well. Cells were washed with serum-free media twice and incubated overnight with DMEM and 1% BSA. Cells were then treated with IL-4 (25 ng/ml) plus GM-CSF (25 ng/ml) or vehicle for 10 min and subjected to the Duolink *in situ* Proximity Ligation Assay as per the manufacturer’s instructions with some modification. Briefly, after the treatment, cells were washed with PBS, fixed with 4% paraformaldehyde for 20 min at 4 °C, and permeabilized with 0.2% Triton X-100 in PBS for 5 min at room temperature. Cells were then blocked with Duolink Blocking Solution in a humidity chamber for 60 min at 37 °C and incubated in a humidity chamber overnight at 4 °C with primary antibodies [anti-rabbit TRPV4 (1:40), and anti-mouse monoclonal Rac1 (1:20)], or with no antibody (control) diluted in Duolink Antibody Diluent. Next day, cells were incubated with probe for 60 min at 37 °C; probes were then ligated for 60 min at 37 °C and were later amplified for 180 min at 37 °C in a humidified chamber. After final washing, coverslips were mounted onto slides with Duolink *in situ* mounting media containing 4′,6-diamidino-2-phenylindole, and analyzed by confocal microscopy (Leica SP5 X Confocal Microscope).

### Adenovirus vector transduction

Adenovirus vector expressing Ad(RGD)-mouse-TRPV4-HIS, Ad(RGD)-GFP, and empty vector control, Ad(RGD)-CMV-null, were obtained from Vector Biolabs (Malvern, PA), with a stock concentration of ∼10^10^ or 10^11^ pfu/ml. For our experiments, after initial standardization, we used 1 × 10^8^ pfu/ml as a working concentration. For transduction, seeded BMDMs were exposed to the working concentration of adenovirus constructs in complete DMEM media, followed by media replacement after 48 h with fresh complete media, and incubated for another 48 h. Cells were utilized for further experiments after 96 h, at which point cells showed maximum forced gene expression based on GFP expression (AdGFP) as confirmed by a fluorescent microscopic analysis (Zeiss Axio Observer microscope).

### G-LISA assay for active Rac1

The G-LISA assay from cytoskeleton was used to assess active GTP-bound Rac1 in cell lysates. For the assay, TRPV4 KO BMDMs were transduced with either Ad-empty vector or Ad-TRPV4 for 72 h in complete DMEM media. BMDMs from WT mice were used as controls with no adenovirus transductions. After 72 h of transduction, all cells were maintained in 1% BSA containing DMEM for 24 h, and then cells were treated with either IL-4 plus GM-CSF (25 ng/ml) or vehicle for 10 min; then cells were resuspended in lysis buffer containing protease inhibitor cocktail (provided in the kit) and lysed on ice using a cell scraper. After clearing the lysate by centrifugation (10,000*g* for 1 min), protein concentrations were measured. The assay was performed using 15 μg of total protein per sample according to the protocol provided by the G-LISA kit manufacturer. Absorbance (490 nm) was measured with a microplate reader (BMG Labtech). Absorbance units for each sample were expressed after subtraction of the background units measured in protein-free lysis buffer.

### Atomic force microscopy

Intracellular stiffness and topography of BMDMs were measured using a JPK Nanowizard 4 AFM (Bruker Nano GmbH, Berlin, Germany) mounted on an inverted optical microscope (Nikon Eclipse TE200, Melville, NY, USA). For quantitative Imaging (QI) measurements of stiffness on living cells, AFM was operated in advanced force spectroscopy–based mode. We used qpBioAC-CI-CB2 cantilevers (Nanosensory, Neuchâtel, Switzerland) with a nominal resonance frequency of 50 kHz in air and partial gold coating on the detector side. A circular symmetric quartz probe with a radius of 30 nm was attached to the cantilever with a spring constant of 0.1 N/m. For the experimental setup, BMDMs (WT, TRPV4 KO, and TRPV4 KO cells transduced with either Ad-Vec or Ad-TRPV4) were seeded on glass bottom, poly-D-lysine–coated 35-mm petri dishes (WPI, Sarasota, FL, USA). After 96 h of transduction, cells were treated with either 25 ng/ml IL-4 plus GM-CSF + vehicle or 25 ng/ml IL-4 plus GM-CSF + Rac1 inhibitor (50 μM) for 24 h. Untreated WT and TRPV4 KO cells without adenovirus transduction were included as controls. For stiffness measurements, cells were kept in PBS buffer at 37 °C, 5% CO_2_, and imaged by acquiring QI maps of 128 × 128 pixels. For all quantitative imaging, a total of 10 cells per condition, 3 to 4 areas per cell, were scanned by applying an imaging setpoint of 0.5 nN. Before measurements, the sensitivity and spring constant of each cantilever was individually calibrated. JPK Data Processing software was used for the calculation of the Young’s modulus (stiffness) by fitting a Hertz contact mechanics model to the generated force curves. For high-resolution imaging, BMDMs were fixed with 3% paraformaldehyde after 24 h of treatment with or without IL-4 plus GM-CSF and Rac1 inhibitor and imaged by QI at a resolution of 512 × 512 pixels.

### Intracellular Ca^2+^ influx measurement

A FLIPR calcium 6 Assay Kit was used to record changes in intercellular Ca^2+^ influx. After seeding on 96-well plates, TRPV4 KO BMDMs were transduced with Ad-TRPV4 expression constructs or Ad-Vec controls for 48 h. BMDMs were washed and incubated for 45 min with FLIPR kit reagent (Calcium 6 dye in 1x HBSS solution containing 20 mM Hepes and 2.5 mM probenecid) at 37 °C and then transferred to the FlexStation 3 System for recording Ca^2+^ influx. During the experiment, GSK1016790A (TRPV4-specific agonist) solution was used as a stimulus to induce Ca^2+^ influx, which was recorded by measuring ΔF/F (Max-Min). Data are shown as relative fluorescence units (37).

### FBGC generation

BMDMs were seeded on Permanox plastic slides (Lab-Tek chamber slides; Nunc, Grand Island, NY) at a density of 1 × 10^5^ cells per well. Cells were maintained for 4 to 7 days in 10% FBS containing DMEM, and IL-4 plus GM-CSF (25 ng/ml) were added on alternate days; cultures were grown in the presence or absence of the Rac1 inhibitor (2, 10, or 50 μM) until cell fusion was maximal (19). Slides were fixed with 4% paraformaldehyde followed by staining with 10x Giemsa solution to confirm generation of multinucleated FBGCs. Five images per well were captured for each condition, and the numbers of giant and single-cell nuclei were counted (Zeiss Axio Observer microscope). The percentage of BMDMs involved in fusion was determined from the number of giant cell nuclei (>5 nuclei) divided by the number of total nuclei per field (19).

### Statistical analysis

All data are expressed as means ± SEM unless otherwise indicated. Statistical analysis was performed using GraphPad Prism software. Student’s *t* test was used for two-group comparisons, and one-way analysis of variance (ANOVA) was used for comparisons between more than two groups. Values of *p* ≤ 0.05 were considered statistically significant.

## Data availability statement

All the data are in the manuscript.

## Conflict of interest

The authors declare that they have no conflicts of interest with the contents of this article.
